# Temperature dependence of time-resolved photoluminescence in closely packed alignment of Si nanodisks with SiC barriers

**DOI:** 10.1186/1556-276X-8-223

**Published:** 2013-05-10

**Authors:** Takayuki Kiba, Yoshiya Mizushima, Makoto Igarashi, Chi-Hsien Huang, Seiji Samukawa, Akihiro Murayama

**Affiliations:** 1 Graduate School of Information Science and Technology, Hokkaido University, Kita 14, Nishi 9, Kita-ku, Sapporo, 060-0814, Japan; 2 Institute of Fluid Science, Tohoku University, 2-1-1 Katahira, Aoba-ku, Sendai, 980-8577, Japan; 3 Japan Science and Technology Agency, CREST, 5 Sanbancho, Chiyoda, Tokyo, 102-0075, Japan; 4 Current address: Department of Electronic Engineering, Chang Gung University, Kwei-Shan, Tao-Yuan, 333, Taiwan

**Keywords:** Time-resolved photoluminescence, Si nanodisks, Neutral beam etching, Bio-nano-templates

## Abstract

We study the temperature dependence of time-resolved photoluminescence (PL) in closely packed alignment of Si nanodisks (NDs) with SiC barriers, fabricated by neutral beam etching using bio-nano-templates. The PL time profile indicates three decaying components with different decay times. The PL intensities in the two slower decaying components depend strongly on temperature. These temperature dependences of the PL intensity can be quantitatively explained by a three-level model with thermal activation energies of 410 and 490 meV, depending on the PL components. The activation energies explain PL quenching due to thermal escape of electrons from individual NDs. This thermal escape affects the PL decay times above 250 K. Dark states of photo-excited carriers originating from the separate localization of electron and hole into different NDs are elucidated with the localization energies of 70 and 90 meV. In contrast, the dynamics of the fastest PL decaying component is dominated by electron tunneling among NDs, where the PL intensity and decay time are constant for temperature.

## Background

Attractive interdisciplinary research areas between electronic and photonic materials have been developed by modern semiconductor nanotechnology. Si nanostructures are particularly important because solar cells using Si have widely been investigated [1,2], and optical interconnections among integrated Si circuits have also been proposed by developing Si-based photodiodes and optical modulators [3,4]. Therefore, many types of Si nanostructures, such as nanocrystals (NCs), nanodots, and porous nanostructures, were reported by employing various fabrication processes [5-14]. Moreover, fabrication processes of the Si nanostructures using ‘top-down’ lithography techniques were strongly motivated for the purpose of applying the Si nanostructures to electronic and photonic devices.

We have recently proposed a fabrication process of Si nanodisk (ND) arrays, where the Si NDs are formed by damage-free neutral beam (NB) etching for Si thin films covered with etching masks of Fe nanoparticles which are regularly aligned by bio-protein engineering [15-20]. This fabrication process using the bio-templates enables us to prepare closely packed high-density Si NDs with the intentionally designed precise size and spacing in a nanometric scale with flexible film stacking. We have also observed intense photoluminescence (PL) emissions in a visible light region with fast decay times ranging from 10 ps to 2 ns [20]. The fast decaying PL characteristics reflect the dynamics of photo-excited carriers in this high-density Si ND array system, in which wavefunctions of photo-excited carriers overlap among Si NDs to some extent, and the carriers can transfer among the NDs [20]. Photo-generated or electrically injected carriers need to be effectively transferred among Si NDs for the optical applications to solar cells or light-emitting diodes. The spatial transfer of the carriers in nanostructures can also be affected by thermal effects, such as thermal hopping or escape. Therefore, in this paper, we investigate the detailed temperature dependence of time-resolved PL and the related carrier dynamics in these high-density Si ND arrays. Different types of PL quenching mechanism can be identified, and the activation energies for the PL thermal quenching are deduced from the temperature dependences of the PL intensity. The difference of the activation energy between different decaying PL components supports the existence of different wavefunctions of the carriers in this high-density ND system. The PL quenching phenomena elucidated in this study will give us useful information about the dynamics of photo-excited carriers, such as carrier separation and transport, when we apply these Si NDs to solar cells and high-speed photonic devices.

## Methods

The high-density (7 × 10^11^ cm^−2^) Si ND arrays were fabricated from polycrystalline Si thin films deposited on thermally oxidized surfaces of Si substrates under ultra-high vacuum. Bio-nano-templates consisting of ferritin supramolecules containing Fe cores were used to prepare two-dimensional closely packed alignments of the Fe cores as etching masks on the surfaces of Si thin films. The size and interspacing of the Fe cores were intentionally designed by protein engineering for the ferritin supramolecules. The Si NDs were fabricated by forming SiO_2_ barriers around the Si NDs masked by the Fe cores using the NB etching and subsequent oxidation processes. Details of the fabrication process are described elsewhere [15-17]. The diameter, thickness, and interspacing distance of the Si NDs mainly used in this study were designed at 10, 4, and 2 nm, respectively, by the abovementioned ferritin-protein engineering. The capping and barrier layers of SiO_2_ were removed with NF_3_ treatment. Then, a 5-nm-thick SiC layer was finally deposited on the Si ND array under a high vacuum by sputtering.

The samples of the Si ND array were placed on a cold finger cooled by a closed He compressor in a vacuum cryostat with quartz windows. The time-resolved PL spectra were observed at various temperatures by combining the excitation of second harmonic femtosecond pulses with the wavelength of 400 nm, pulse width of 150 fs, and repetition rate of 76 MHz of a mode-locked Ti-sapphire laser, with the detection of a synchroscan streak camera (Hamamatsu Photonics, Hamamatsu, Japan). A spot diameter of the laser light focused on the sample surface was 100 μm. The excitation power density was 8.4 mJ cm^−2^. The number of electron–hole pair generated per one ND was calculated to be less than 1, taking the sheet density of ND into account. Therefore, the multiple exciton generation or Auger process were not induced. The time width of the instrumental response curve was less than 15 ps, and the time resolution of 5 ps was obtained after deconvolution with the instrumental response.

## Results and discussion

**Figure 1:**
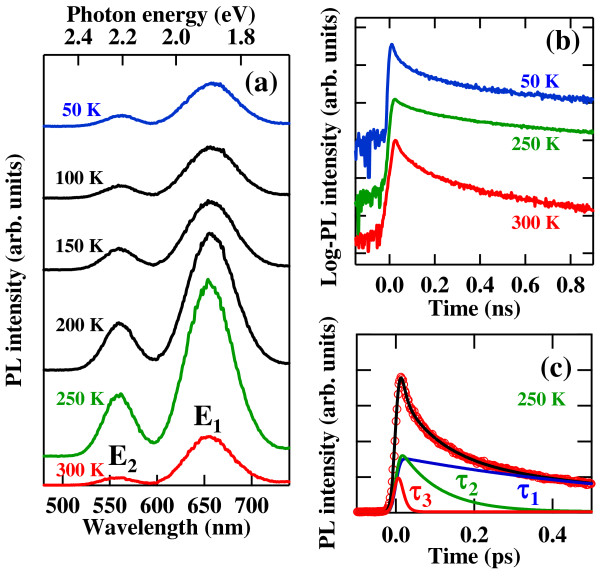
**Time-integrated PL spectra, transient PL, and typical fitting result.** Time-integrated PL spectra in the high-density Si ND array with SiC barriers at various temperatures (**a**). PL time profiles (log-scaled and vertically shifted) of the *E*_1_ emission band indicated in (a) from the Si ND array for various temperatures (**b**). Typical fitting result of the PL time profile at 250 K using a triple exponential function, where the PL time profile is deconvoluted with an instrumental response function (**c**). A bold black line shows a fitting calculation, and each decaying component resolved is shown by a narrow line.

Time-integrated PL spectra of the Si ND array at various temperatures are shown in Figure 1a. PL emission bands with the wavelengths of 655 nm (1.89 eV, *E*_1_ band) and 564 nm (2.22 nm, *E*_2_ band) are visible for the whole temperature range. The observed PL cannot be attributed to the indirect bandgap emission affected by a quantum confinement effect, which was often reported in small Si NCs with diameters of 2 to 5 nm. These confined emission energies increased up to 1.7 eV, as reported in Si NCs with the diameter of 2.5 nm [6]. The optical bandgap energy of our Si ND system with the thickness of 4 nm and diameter of 10 nm has been calculated to be *ca*. 1.5 eV from the one-band Schrodinger equations with classic envelope function theory [19]. However, in our case, the PL peak energy is markedly higher than these energies. Moreover, as described later, decay times of the observed PL are ranging from 10 ps to 2.0 ns, which are much shorter than those in the microsecond-scale characteristic for the indirect bandgap recombination of carriers or defect-related emissions. There are several reports for surface-related emissions in the visible light region, which have been confirmed by PL measurements of samples with different surface treatments [10]. The spectral widths of the PL bands are less than 200 meV. The spectral linewidths of single Si nanocrystals were reported to be 100 meV or more [5,21], which were also dependent on the fabrication method and surface conditions. In our case, the size of the Si ND was precisely controlled by the diameter of the Fe core formed in a cavity of the ferritin molecule. The size uniformity of 8% was confirmed from the statistical analysis of SEM images [17]. Therefore, an effect of inhomogeneous broadening due to the size distribution on the PL spectral shape is estimated not to be significant. This estimation is supported by a fact that no remarkable spectral diffusion, which is a time-dependent redshift of the PL spectral energy, was observed for both PL bands in the time-resolved PL spectra. Time-dependent redshifts due to thermal hopping of carriers or energy transfer were frequently observed in systems of high-density quantum dots with significant size distributions.

Temperature dependences of the spectral shape and energy were not seen. Both PL bands exhibit similar temperature dependences of the intensity. The PL intensity of the *E*_2_ band is much weaker than that with the SiO_2_ barrier, which was previously reported [22]. Therefore, we consider that this *E*_2_ band originates from oxygen-related surface or interface states of the Si NDs, and we would like to discuss mainly about the *E*_1_ emission. In the low-temperature regime below 150 K, the PL intensity is almost constant. The intensity increases toward 200 K and peaks at a maximum around 250 K. The PL intensity then decreases monotonically with further increasing temperature. Quantitative discussion about these temperature dependences of the PL intensity will be made later.

The transient PL for the *E*_1_ band emission as a function of temperature in the Si ND array is shown in Figure 1b. The temporal evolution of each PL profile cannot be expressed by a single exponential function. The best fit was obtained typically using a triple exponential function as shown in Figure 1c, which is common for all array samples of the high-density Si NDs. From this fitting, we have identified three PL decaying components with different time constants *τ*_1_ = 770 ps, *τ*_2_ = 110 ps, and *τ*_3_ = 15 ps, respectively, for this case at 250 K as an example. Several papers have demonstrated ultrafast PL in a sub-picosecond region for Si NCs by means of up-conversion PL. The ultrafast emission ranging 2.0 to 2.4 eV was observed, which was attributed to the pseudodirect gap emission from the core states of Si NCs [11,12]. In contrast, the PL components observed in our samples show time constants ranging from 10 ps to 1 ns, where values are much higher than those of the above pseudodirect gap emissions. Therefore, the most probable origin of the *E*_1_ emission is emissive surface states weakly located at the interfaces of Si NDs. Dhara and Giri have reported the PL emission with the wavelength of about 600 nm with decay times of several nanoseconds [13]. They assigned this PL to the quasi-direct bandgap emission in heavily strained Si NCs because of their unique preparation of the NCs by milling. Sa'ar reviewed recent developments in the PL studies of various Si nanostructures and suggested that neither quantum confinement model nor surface chemistry model can solely explain the entire spectrum of emission properties [14].

The three PL components with different decay times imply three different types of emissive sites in the present ND array. We assigned these three decaying components from the disk density and excitation power dependences of the PL decay time and intensity [20]. The emission with the slowest decay time *τ*_1_ on the order of 1 ns was interpreted by electron–hole pairs or excitons localized at individual NDs, because this PL component was dominant in the case of low-density dispersive NDs with the disk interspacings larger than 40 nm. The emission with the decay time *τ*_2_ was understood by recombination of an electron–hole pair or exciton not strongly localized in each ND, where each wavefunction of the carrier spreads over neighboring NDs to some extent due to periodic regular alignment of the ND separated by ultrathin potential barriers. The fastest PL component with *τ*_3_ was attributed to the recombination which was strongly affected by the electron tunneling among the NDs. In other words, this fastest PL was quenched by the electron transfer. The latter two faster PL components appeared only at high excitation densities in the high-density ND arrays. The interpretation of the electron transfer was also supported by the conductance measurement for sister samples [23].

**Figure 2:**
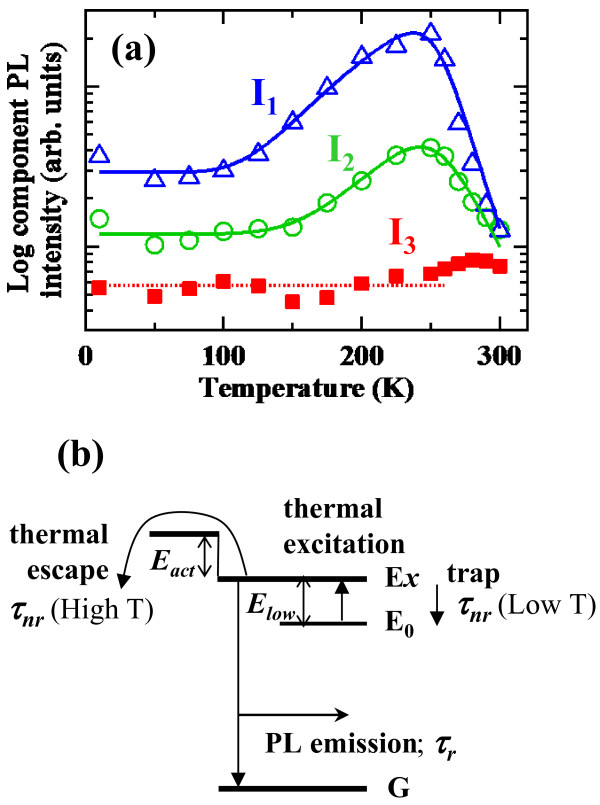
**Time-integrated PL intensities. ***Ι*_1_ (an open blue triangle), *Ι*_2_ (an open green circle), and *Ι*_3_ (a closed red square) of the individual decaying components with the decay times *τ*_1_, *τ*_2_, and *τ*_3_, respectively, as a function of temperature in the Si ND array with the SiC barrier (**a**). Solid blue and green lines are calculations using a three-state model. A dotted red line is the guide for the eyes. A schematic illustration of the three-level model used in the analysis for the temperature dependences of PL intensities of time-resolved *I*_1_ and *I*_2_ components (**b**).

The time-integrated PL intensities of the three decaying components were deduced by fitting the PL decay curves with the triple exponential function. The PL intensities are plotted as a function of temperature in Figure 2. As can be seen, time-integrated intensities of the two slower decaying components (*I*_1_ and *I*_2_, corresponding to the PL components with the decay times *τ*_1_ and *τ*_2_) depend strongly on temperature, while the fastest decaying component (*I*_3_ with *τ*_3_) is almost constant for temperature. We analyzed these temperature dependences of PL intensities of the *I*_1_ and *I*_2_ components by a thermal quenching model taking an existence of ‘middle state’ into account [24]. In our calculation, we assumed that the time-integrated intensity of the observed PL was equivalent to that measured by the steady-state excitation because the PL decay times in the present Si ND system are below 2 ns. In this model, we considered three levels schematically shown in Figure 2b. The emissive excitonic level denoted by *E*_*x*_ is assumed to exist between the barrier level for thermal escape of photo-excited carriers from individual NDs and the lower-energy level *E*_0_. This *E*_0_ level is possibly due to localization at trap states formed by spatial displacements of wavefunctions of an electron and hole in the ND system. The electronic states in the Si NDs can largely be affected by the interfacial bonding states of Si atoms. Therefore, radiative interfacial states (*E*_*x*_) and deeper trap levels (*E*_0_) can be formed. The PL intensity from this middle state is basically proportional to the number of electron–hole pair or exciton at this level and thus dependent on a thermal escape rate beyond the barrier as well as on a thermal excitation rate from the lowest trap level. In this case, the PL intensity can be described as follows:

(1) $$I\left(T\right)=I\left(0\right)\frac{1+D\exp\left(-E_{\mathrm{low}}/k_{b}T\right)}{1+C\exp\left(-E_{\mathrm{act}}/k_{b}T\right)}$$

where *E*_act_ and *E*_low_ are activation energies for the thermal escape and thermal excitation, respectively. *C* and *D* are proportionality factors. The calculations using Equation 1 are fitted to experimental values and shown by solid lines in Figure 2a.

The *E*_act_ values, which express PL quenching slopes in the high-temperature region, were determined to be *E*_act1_ = 490 meV and *E*_act2_ = 410 meV for the time-resolved *I*_1_ and *I*_2_ components, respectively. The *E*_act_ values represent the energy difference between the emissive state *E*_*x*_ and the transfer channel among NDs for thermally activated electron hopping. These values of *E*_act1_ and *E*_act2_ agree well with the barrier height of 0.44 eV for the electron, taking into account the SiC bandgap energy of 3.0 eV obtained from reflectance measurements of a thick SiC film fabricated by the same deposition method and the PL energy of 1.9 eV for the Si NDs. The valence band offset is assumed to be 0.6. The valence band offset for the Si/SiC interface has not been known as far as we know, but that of the Si/SiO_2_ has been recently determined to be 0.6 [25]. Therefore, we interpret that the obtained *E*_act_ values show a potential height for the electron at the Si ND/SiC interface. The difference in the above two activation energies (*E*_act1_ − *E*_act2_ = 80 meV) indicates the energy difference between the two emissive states showing the different decay times of *τ*_1_ and *τ*_2_, if we assume that the potential height responsible for the thermal escape is identical for both PL components. On the other hand, the *E*_low_ values, which describe negative quenching slopes of the gradual increases in the PL intensity at low temperatures, are obtained to be *E*_low1_ = 70 meV and *E*_low2_ = 90 meV for the time-resolved *I*_1_ and *I*_2_ components, respectively. At low temperatures, the electron and hole responsible for the *I*_1_ emission are separately trapped at different shallow potential minima within each ND, and the recombination rate significantly decreases. These spatially isolated electron and hole are thermally depopulated within the ND and can recombine at higher temperatures, which results in increases in the PL intensities. This lowest energy level is efficiently not emissive, but the carriers are not extinct via defect-related non-radiative centers because the thermally excited carriers from these states emit strong PL at higher temperatures.

The electron and hole responsible for the *I*_2_ emission can be localized at different NDs in the low-temperature regime and then the electron and hole pair is spatially separated. Therefore, the recombination probability decreases significantly (non-emissive). Increasing the temperature, the electron and hole are thermally excited to the free-like state (emitting *I*_2_), and the recombination of the electron and hole can take place again. We find that the *E*_low2_ value indicating the activation (localization) energy of the *I*_2_ emission at low temperatures agrees well with the energy difference between *E*_act1_ and *E*_act2_; *E*_act1_ (490 meV) − *E*_act2_ (410 meV) = 80 meV (*E*_low2_ = 90 meV). From this correspondence, we attribute the gradual increase in the *I*_2_ emission at the low-temperature region to the thermal excitation of the carriers from the dark state, where the electron and hole are localized in different individual NDs, to the free-like state among neighboring NDs.

**Figure 3:**
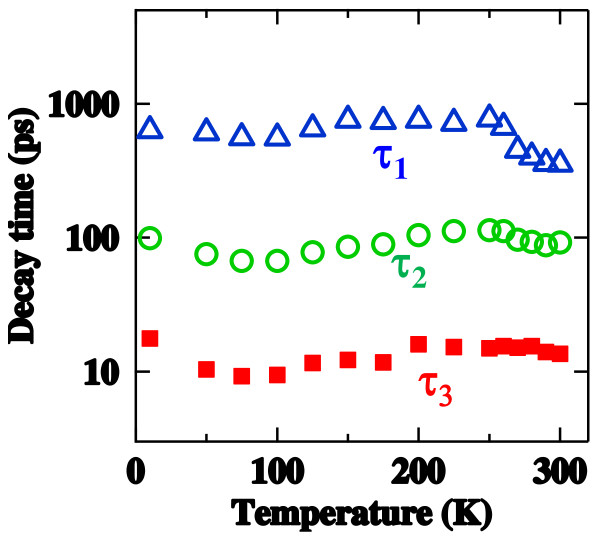
**PL decay times. ***τ*_1_ (an open blue triangle), *τ*_2_ (an open green circle), and *τ*_3_ (a closed red square) as a function of temperature for the Si ND sample with the SiC barrier.

Figure 3 shows PL decay times of the three PL components as a function of temperature. The decay times of *τ*_1_ and *τ*_2_ decrease monotonically with increasing temperature in the high temperature region above 240 K. This temperature-induced lifetime shortening coincides well with the abovementioned thermal quenching due to the electron escape from individual NDs through the transfer channel. Therefore, we conclude that the PL decay characteristics at the high-temperature region are significantly affected by the thermal escape of electrons. In contrast, the PL decay time of *τ*_3_ is almost constant for temperature. This fact infers that electron tunneling through thin barriers play a significant role for the decay characteristics of this fastest PL component rather than the thermal hopping. The picture of ultrafast tunneling of the electron has been discussed in our recent paper and is supported by an experimental fact that the fastest PL component with *τ*_3_ appears only when high-density excitations are made for the dense ND system [20]. The electron tunneling process will be important when we consider applications of superlattices composed of the present high-density Si NDs to solar cells with high efficiencies because a photo-excited electron–hole pair can be immediately separated by this tunneling process before the radiative recombination takes place. Further efforts to enhance the tunneling process will be performed by designing proper barrier materials and the spatial alignment of NDs.

**Figure 4:**
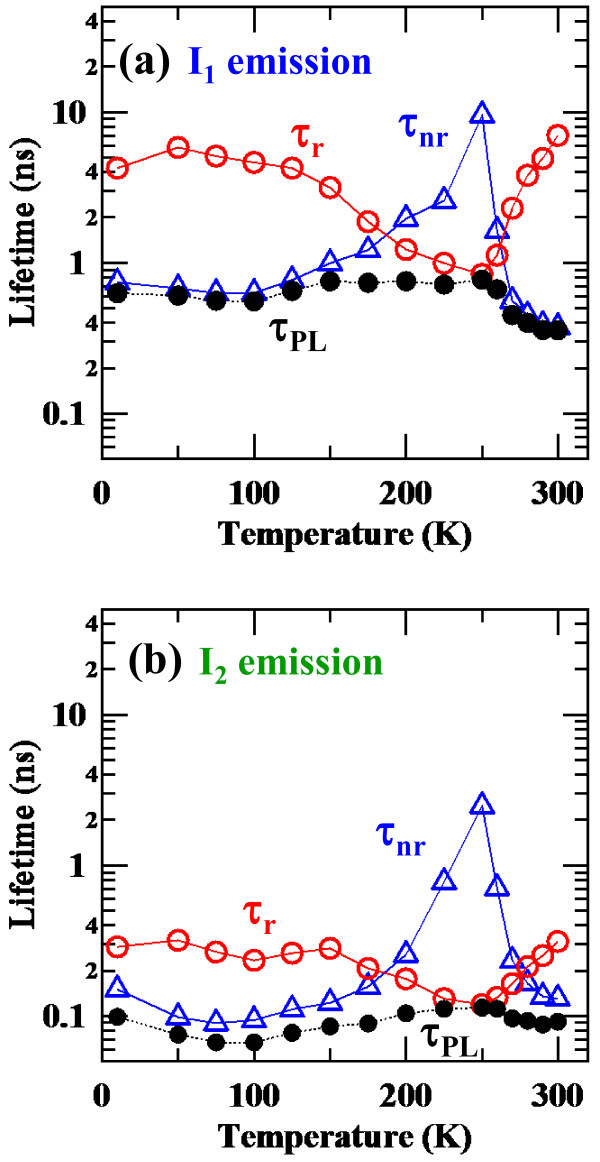
**Radiative lifetime *τ***_**r **_**(an open red circle) and non-radiative lifetime *τ***_**nr **_**(an open blue triangle).** Calculated using Equations 2 and 3 as a function of temperature for the *I*_1_ (**a**) and *I*_2_ (**b**) PL components. PL decay time *τ*_PL_ (a closed black circle) is also plotted.

Finally, we discuss about the temperature dependences of the PL decay time based on the abovementioned non-radiative decaying processes possibly caused by the thermal quenching beyond the barriers and energy relaxation to the localization or trap states. The PL decay times of the *I*_1_ and *I*_2_ components can be separated into a radiative lifetime *τ*_r_ and non-radiative lifetime *τ*_nr_ if we assume that the internal quantum efficiency of each PL component is 1 at the temperature showing the maximum PL intensity. The *τ*_r_ and *τ*_nr_ were calculated using the following equations:

(2) $$\tau_{r}\left(T\right)=\tau_{\mathrm{PL}}\left(T\right)\frac{I_{\max}}{I\left(T\right)}$$

(3) $$\tau_{\mathrm{nr}}\left(T\right)=\tau_{\mathrm{PL}}\left(T\right)\frac{I_{\max}}{I_{\max}-I\left(T\right)}$$

where *τ*_PL_ is the PL decay time measured, and *I* and *I*_max_ are the PL intensity at a certain temperature *T* and the maximum PL intensity, respectively. If the quantum efficiency at the temperature showing the maximum PL intensity is smaller than 1, absolute values of both the *τ*_r_ and *τ*_nr_ varies. However, the trends of the temperature dependences of the *τ*_r_ and *τ*_nr_ should be similar because the PL intensity shows non-monotonic temperature dependence. The τ_r_ and *τ*_nr_ lifetimes deduced for the *I*_1_ and *I*_2_ components are plotted as a function of temperature in Figure 4a,b, respectively, together with the measured *τ*_PL_.

At the lower temperature region below 200 K, the *τ*_nr_ value decreases with decreasing temperature, and the *τ*_PL_ becomes dominated by the *τ*_nr_. This trend can be understood by the existence of non-emissive localized or trap states as discussed above. The *τ*_nr_ value increases toward the maxima with increasing temperature because of the thermal excitation of the carriers from the localized or trap levels to the emissive ones. In contrast, in the high-temperature regions toward room temperature, the *τ*_nr_ decreases with increasing temperature because of the thermal escape from the emissive level beyond the barriers. These PL dynamics for the two slower decaying PL components of *I*_1_ and *I*_2_, expressed by the temperature dependences of the *τ*_r_ and *τ*_nr,_ agree well with the thermal quenching and excitation processes elucidated by the temperature dependences of intensities of these PL components.

## Conclusions

We have studied temperature dependences of time-resolved PL in the two-dimensional high-density Si ND arrays fabricated by NB etching using bio-nano-templates, where the PL time profiles with various temperatures are fitted by triple exponential decay curves. We find that the time-integrated PL intensities in the two slower decaying components depend strongly on temperature, which is attributed to PL quenching due to thermal escape of electrons from emissive states of individual NDs in addition to thermal excitations of carriers from localized or trap states in the individual NDs to the emissive ones. The temperature dependences of the PL intensity were analyzed by the three-level model. The following thermal activation energies corresponding to the thermal escape of the electron are obtained to 410 and 490 meV, depending on the PL components. In addition, we find dark states of photo-excited carriers, which can be attributed to the separate localization of the electron and hole into different NDs with the localization energies of 70 and 90 meV, depending on the PL components. The PL decay times of these two decaying components ranging from 70 to 800 ps are also affected by this thermal escape at high temperatures from 240 to 300 K. The fastest decaying component shows a constant decay time of about 10 ps for various temperatures, in which the decay characteristic is dominated by the electron tunneling among NDs.

## Competing interests

The authors declare that they have no competing interests.

## Authors' contributions

TK and AM conceived the spectroscopic study, participated in its design and coordination, and drafted the manuscript. TK and YM carried out the time-resolved PL measurement and analyzed the data. MI, CH, and SS conceived the fabrication process and participated in its design and coordination. MI and CH fabricated the Si-ND array sample. All authors read and approved the final manuscript.
